# Postoperative infections following silicone implant-based gluteal augmentation

**DOI:** 10.1016/j.jpra.2026.09.017

**Published:** 2026-09-16

**Authors:** Elisa Bertulla, Francesca Maria Scura, Edoardo Raposio

**Affiliations:** a Department of Surgical Sciences and Integrated Diagnostics (DISC), University of Genova, Viale Benedetto XV 6, 16132, Genoa, Italy; b Plastic and Reconstructive Surgery Division, AOM - IRCCS Ospedale Policlinico San Martino, Genoa, Italy

**Keywords:** Gluteal implant augmentation, Gluteoplasty, Infection, Surgical site infection, Implant-related infection

## Abstract

**Background:**

Gluteal augmentation with silicone implants is increasingly performed worldwide, but postoperative infection remains one of the most relevant complications. This complication can significantly affect both the survival of the implants and the overall outcomes for patients. However, evidence regarding incidence, microbiology, risk factors, and management remains heterogeneous.

**Methods:**

A systematic review was conducted according to PRISMA 2020 guidelines. PubMed, Scopus, the Cochrane Library and Latin American and Caribbean Literature in Health Sciences (LILACS) database were searched for studies reporting infectious complications following aesthetic gluteal augmentation with silicone implants. Study quality was assessed using the Joanna Briggs Institute (JBI) critical appraisal tools. The certainty of evidence was assessed using the Grading of Recommendations Assessment, Development and Evaluation (GRADE) approach across the four prespecified outcome domains. Given clinical and methodological heterogeneity, data were synthesized narratively.

**Results:**

Twenty-five studies, including 4937 patients, were included. Most studies were retrospective case series or case reports, with limited comparative evidence. Infection rates could not be reliably estimated because of heterogeneous definitions, incomplete reporting, and variable follow-up. Only a small number of studies provided microbiological findings, with *Staphylococcus aureus* being the most commonly identified bacteria in culture-confirmed infections. Available data were insufficient to determine the influence of implant plane, implant characteristics, or technical modifications on infection risk. Management strategies included antibiotic therapy and implant removal. According to JBI assessment, 15 studies were classified as low risk of bias, 6 as moderate risk, and 4 as high risk. The certainty of evidence was rated as low for infection incidence and very low for the remaining three outcome domains.

**Conclusions:**

While postoperative infections in gluteal implant augmentation seem to be rare, they can lead to serious complications. However, the current evidence is limited due to methodological heterogeneity and inconsistent reporting. Prospective comparative studies using standardized definitions and detailed microbiological and clinical data are required to better guide prevention and management.

## Introduction

Interest in surgical procedures aimed at refining the body contour of and enhancing gluteal aesthetic has increased steadily in recent years, paralleled by growing scientific efforts to define objective anatomical criteria or aesthetic proportion. Foundational work identifying the waist-to-hip ratio as a determinant of perceived attractiveness,^1^^,^^2^ together with cross-cultural analyses of body ideals^3^ has been extended by more recent geometric investigations of the umbilicus,^4^^,^^5^ infragluteal fold,^6^ lateral thigh,^7^ anterior torso,^8^ and buttocks,^9^, ^10^, ^11^, ^12^, ^13^including comparisons with classical artistic canons.^14^ Among these regions, the buttocks occupy a particularly prominent place, reflecting the substantial growth in demand for gluteal aesthetic surgery over the past two decades.

Annual surveys from the International Society of Aesthetic Plastic Surgery consistently show that buttock augmentation is one of the fastest-growing body-contouring procedures worldwide, including both fat grafting and implant-based techniques.^15^^,^^16^ Recent concerns about fat embolism and the risks associated with autologous fat transfer, as pointed out in the ASERF task force report on gluteal fat grafting deaths,^17^ have renewed interest in implant-based augmentation. This method is seen as a more standardized and reliable option, especially for those with limited gluteal soft tissue or who prefeor a more permanent solution.^18^^,^^19^^h^

Augmentation gluteoplasty with solid silicone implants was first systematically described in the 1980s using a submuscular plane,^20^with later modifications describing intramuscular, subfascial, and subcutaneous pockets, each with distinct trade-offs in coverage, stability, and complication risk.^18^^,^^21^^,^^22^ Indications include gluteal hypoplasia, asymmetry, post-traumatic contour deformities, and purely aesthetic requests for increased projection.^19^^,^^23^ The procedure requires meticulous pocket dissection and strict sterile technique, given the recognized vulnerability of implant-based gluteal surgery to infection. Postoperative infection is among the most clinically significant complications of gluteal implant surgery, associated with implant loss, reoperation, and, rarely, systemic sepsis.^21^^,^^22^ The gluteal region may present unique challenges compared with other implant-based aesthetic procedures due to its proximity to the perineum, greater local bacterial colonization, mechanical pressure during sitting, and variable soft-tissue coverage.^22^^,^^23^ Proposed risk factors include smoking, seroma/hematoma formation, oversized implants, subcutaneous placement, and inadequate activity restriction, with variable strength of association across studies.^18^^,^^22^^,^^23^ However, data regarding the microbiology and management of these infections remain fragmented.

Diagnosis can be challenging, as infections may present with an indolent course and biofilm-related features sometimes resembling a simple seroma or delayed healing rather than overt cellulitis.^21^^,^^22^ Management ranges from antibiotic therapy and pocket salvage to mandatory explantation, with no standardized algorithm currently guides treatment decisions. Furthermore, considerable heterogeneity exists in how infection are defined and reported across studies.^22^, ^23^, ^24^

While several systematic reviews have addressed complications of implant-based gluteal augmentation more broadly, covering aspects such as pocket location, incision technique, and overall safety profiles,^25^^,^^26^ infection has typically been reported as one endpoint rather than as the primary focus of investigation. However, considerable variability remains in the reported incidence, diagnostic criteria, and management strategies specific to this complication. To our knowledge, no systematic review to date has focused specifically on infectious complications following gluteal implant augmentation.

With the increasing number of gluteal implant procedures being performed, the clinical significance of postoperative infection, and these gaps in the literature, a systematic review of available literature is needed. This review aims to summarize the incidence, microbiology, risk factors, diagnostic approaches, and management strategies for infections following gluteal augmentation with silicone implants, with the goal of improving the evidence base for prevention and clinical management.

## Patients and methods

### Search criteria

The research was performed on July 25, 2026 using Pubmed, Scopus and Cochrane Library databases, with combinations of terms related to gluteal augmentation procedure with implants. A supplementary search was subsequently performed on Latin American and Caribbean Literature in Health Sciences (LILACS) database on September 1, 2026 to identify potentially eligible studies that may not have been captured by the databases included in the original search. The search string was adapted to include Spanish terms, while the same eligibility criteria and study selection process used in the original search were applied. In addition, backward and forward citation searching was performed for the included studies to identify further potentially eligible publications. Two authors independently screened titles and abstracts, followed by full-text assessment of potentially eligible studies. Any disagreements arising during the screening process were resolved through discussion and consensus.

The full search strategies for all databases are provided as Appendix I. This systematic review was conducted according to the Preferred Reporting Items for Systematic Reviews and Meta-Analysis (PRISMA) guidelines 2020.^27^

The research question was formulated according to the PICO framework: “P: Patients undergoing aesthetic gluteal augmentation with implants; (I): Gluteal augmentation performed using silicone implants. (C): Not applicable (or, where available, comparison between different implant types, surgical approaches, perioperative protocols, or infection management strategies). (O): Postoperative infectious complications, including incidence, microbiological profile, risk factors, diagnostic methods, management strategies.

This manuscript was registered on PROSPERO (ID: CRD420261461081).

### Selection criteria

Eligibility criteria were predefined before the literature search to minimize selection bias.

#### Inclusion criteria

- Studies involving human participants
- Studies including patients who underwent aesthetic gluteal augmentation with implants
- Studies reporting infectious outcomes (presence or absence of postoperative infections)

#### Exclusion criteria

- Reviews, systematic reviews, meta-analyses editorials, expert opinions, commentaries, study protocols, book chapters, conference abstracts or proceedings, and letters to the editor, and studies for which the full text was not available.
- Studies involving patients undergoing gluteal reconstruction following trauma, oncologic resection, congenital abnormalities, or other reconstructive indications, and revision/secondary gluteoplasty.

Revision and secondary gluteoplasty procedures were excluded to specifically evaluate the risk of infection associated with primary gluteal implant surgery and to minimize potential confounding related to previous surgical interventions.

- Studies analyzing gluteal augmentation performed with autologous fat grafting, injectable fillers, or other non-implant techniques
- Studies in which data specific to implant-based gluteal augmentation could not be extracted separately from data on other gluteal augmentation procedures.

Data extraction was performed independently by two reviewers, with any disagreements resolved through discussion until consensus was reached. Data extraction included study characteristics, population, implant characteristics, surgical technique, implant plane, infection incidence, microbiological findings and management strategies.

The methodological quality of included studies was assessed by two independent reviewers using the Joanna Briggs Institute (JBI) Critical Appraisal Checklists for Case Series, Case Reports and Cohort Studies, according to the study design.^28^, ^29^, ^30^ Each item was scored as “Yes” (1 point), “No” (0 points), or “Unclear” (0 points). A total quality score was calculated as the proportion of “Yes” responses. Studies were classified as low risk of bias (≥70%), moderate risk of bias (50–69%), or high risk of bias (<50%).

The certainty of evidence was assessed using the GRADE^31^approach for the four prespecified outcome domains: infection incidence, pathogen distribution, implant plane and infection, and management of infection. The overall certainty of evidence for each outcome was classified as high, moderate, low, or very low. As the included evidence was predominantly observational, the initial certainty was rated as low. Assessment was conducted across the GRADE domains: risk of bias, inconsistency, indirectness, imprecision, and publication bias. Evidence was downgraded by one or two levels when serious or very serious concerns were identified for any of these domains.

Due to the substantial clinical and methodological heterogeneity among included studies, including differences in surgical techniques, implant planes, outcome definitions, follow-up duration, and study designs, quantitative synthesis using meta-analysis was considered inappropriate. Therefore, findings were synthesized narratively.

## Results

A total of 25 studies were included,^32^^,^^33^^,^^23^^,^^21^^,^^34^, ^35^, ^36^, ^37^, ^38^, ^39^, ^40^, ^41^, ^42^, ^43^, ^44^, ^45^, ^46^, ^47^, ^48^, ^49^, ^50^, ^51^, ^52^, ^53^, ^54^ comprising 4937 patients who underwent gluteoplasty with implants. The study selection process is summarized in the PRISMA flow diagram (Fig. 1).

**Fig. 1:**
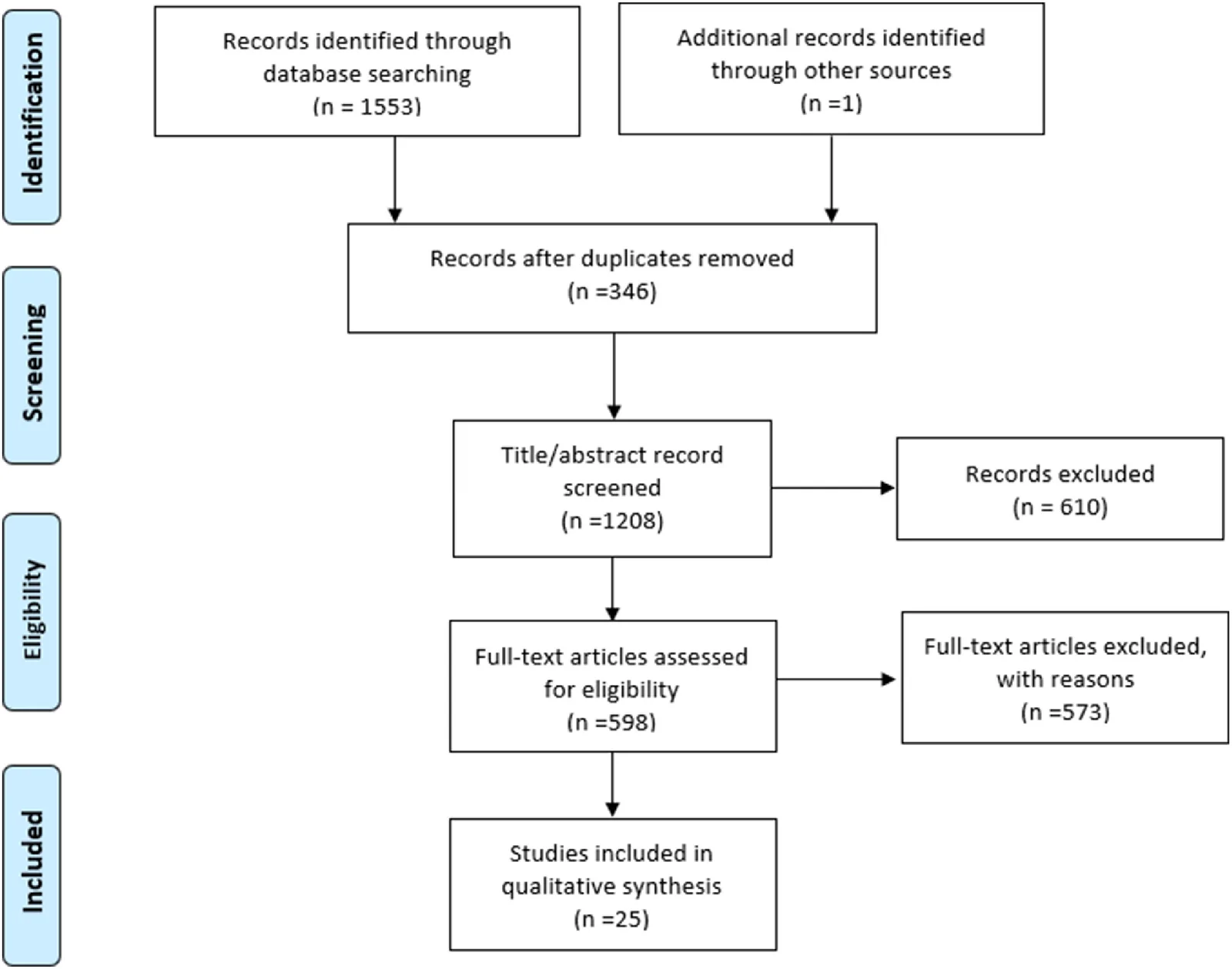
PRISMA flow-diagram.

Characteristics of the included study are summarized in Table 1.

**Table 1:** Summary of the included study.

Authors and year	Study Design	Population	No. Of Patients	Age range	Type of Implant	Implant plane/Technique	Intraoperative Irrigation pocket	Follow up (months)	No. of Infections (%)	Characteristics of infection	Treatment
Danilla et al., 2019^32^	Retrospective case series	1 male, 18 females	19	21–50	Oval anatomical silicone implants	IM (XYZ technique)	NR	NR	0	-	-
Gonzalez 2004^33^	Retrospective case series	7 males, 739 females	746	16–64	743 Smooth implants, 3 texturized. 72& round, 28% oval	IM (XYZ technique)	NR	NR	3 (0,4%)	NR	Culture-directed antibiotic therapy, saline- solution irrigation
Mofid et al., 2013^23^	Cross-sectional surgeons survey	19 surgeons	2226	NR	12 /19 surgeon in their practice used textured implants (63%), 10/19 (52%) AART implants*	IM plane preferred by 13/19 surgeons (68.4%).	Used by 16/19 surgeons (84%)	NR	80 (3.6%) minor infections; major infection: NR (included among 85 implant removals for major infection, chronic pain, and chronic seroma).	NR	Minor infection was managed without implant removal. Major infections required implant removal.
Senderoff 2011^21^	Retrospective case series	27 males, 174 females	200	NR	Silicone implants	154 SF; 46 IM plane	Yes (iodopovidone solution)	32 (mean)	15 /400 (3.8%) implant infections; 13/200 (6.5%) patients with infections. 3 infections occurred in the IM group and 10 occurred in the SF group	*Staphylococcus aureus* (11 patients) *E. coli (1* patient) In 1 patient no fluid was available for culture.	Implant removal (11/200 patients) (5.5%). Antibiotics alone (2 patients).
Park et al., 2016^34^	Retrospective case series	125 females and 5 males	130	NR	Oval smooth silicone implants	IM	NR	NR	2 (1.5%)	NR	NR
Mezzine et al., 2015^35^	Surgeon- survey	13 surgeons	538	NR	16% of surgeons used textured implants, 83.3% smooth implants	IM	NR	NR	1.4% of major infections; 0.3% minor infections	NR	Implant removal for major infections. Treatment of minor infection was not reported
Petit et al., 2022^36^	Retrospective case series	98 females, 2 males	100	19–59	Smooth round implants	SM	NR	28–39	0	-	-
Salamat et al., 2015^37^	Case series	NR	3	NR	AART implants	IM with use of Retention Sutures removed after 3 weeks	NR	6	0	-	-
Triana et al., 2021^38^	Retrospective case series	NR	A total of 4670 patients. Of these 423 gluteal implants	NR	NR	IM	NR	NR	3 (0,06%)	NR	NR
Alvarez-Trejo et al., 2026^39^	Retrospective case series	Females	17	NR	Round silicone implants	IM	NR	24 (mean)	0	-	-
Ramirez-Sanchez et al., 2025^40^	Case series	Patients (females) with postoperative infections after cosmetic surgery	A total of 6. Of these 2 gluteal implants	22 and 26	NR	Replacement of implants (plane not reported)	NR	NR	2	*Cutibacterium avidum*	Surgical debridement, antibiotic therapy and implants removal
Badin et al.,2007^41^	Retrospective case series	Females	28	20–52	Smooth, round, high-cohesion silicone silimed implants	Endoscopically assisted SM	NR	NR	1 wound infection (3.5%), 1 subcutaneous site infection (3.5%), 0 implant infection	NR	NR
Andrade et al., 2017^42^	Retrospective case series	Females	12	23–49	NR	IM	Cephazolin and gentamicin	17.42(mean)	0	-	-
Aboudib et al., 2012^43^	Retrospective case series	NR	103	19–59	NR	IM	NR	NR	1 (0.97%)	NR	Implant removal
Alperovich et al., 2007^44^	Case report	Pregnant woman	1	32	NR	NR	NA	NA	1	Late infection manifesting 27 months after procedure, with abscess, fever, leukocytosis (preceded by a wound dehiscence) Microbiological finding: *Pseudomonas Aeruginosa*	Drainage of the abscess, antibiotic therapy and implant removal
Vergara et al., 2003^45^	Retrospective case series	Femaless	160	18–65	Textured implants; 104 (65%) silicone gel implants; 56 (35% silicone elastomer implants	IM	NR	NR	0	-	-
Ibarra-Hurtado et al., 2024^46^	Case series	Females	14	42.5 (mean)	High-profile cohesive silicone gel implants	Endoscopically assisted- IM	NR	20.9 (mean)	0	-	-
Stojicic et al., 2023^47^	Case report	Female	1	39	NR	IM	NA	NA	1	Manifesting with dehisced wound with purulent secretion in a concomitant SARS-coV-2 infection. Diagnosis of necrotizing fasciitis due to *E. coli*	Surgical debridement, antibiotic therapy and implants removal
Serra et al., 2012^48^	Comparative study (prospective and retrospective cohorts)	Females (group A: gluteoplasty with modified technique prospectively enrolled); group B classic technique retrospectively analyzed)	40 (20 in group A; 20 in group B)	Group A: 20–47; group B: 22–51	Smooth-surface cohesive gel implants with spherical or anatomical bases	IM with modified wound management technique	NR	NR	0 in group A; 1 in group B (5%).	NR	NR
Hidalgo et al., 2006^49^	Retrospective case series	Females	97	23–55	75 silicone gel implants; 22 silicone elastomer implants. Both round smooth-surface	SM	NR	NR	3 infections in silicone gel implants (4%) (2 superficial wounds and 1 submuscular); 0 in silicone elastomer implants	NR	Superficial wound infections were treated with antibiotics based on culture findings; submuscular infection with implant removal and antibiotic therapy (re-implantation after 6 months)
Azevedo t al., 2012^50^	Retrospective case series	Females	A total of 8 (4 implants + liposuction excluded; 4 only implants included)	28–50	Silicone elastomer implants	IM (XYZ technique)	Yes, cefalotine	NR	0	-	-
Carvalho et al. 2012^51^	Retrospective case series	16 females and 2 males	18	25–40	Silicone elastomer implants	IM	NR	NR	0	-	-
Castilho et al., 2017^52^	Retrospective case series	26 females and 3 males	29	22–64	Silicone elastomer implants	IM (XYZ technique)	NR	NR	0	-	-
Tamayo Carbon et al., 2023^53^	Prospective case series	Females	25	31–40	Silicone gel implants	IM	NR	NR	0	-	-
Landfald et al., 2025^54^	Case report	Female	1	21	NR	IM	NR	NR	Early infection presented with pain, swelling and systemic symptoms	*Coagulase-negative Staphylococcus*	EV antibiotic therapy, surgical wound management and implant removal

NR = not reported; AART = manufacturer of gluteal implants (Reno, Nevada, USA); IM = intramuscular; SF = subfascial; SM = submuscular; XYZ = specific surgical technique.

⁎ Categories are not mutually exclusive.

Because the primary studies did not provide sufficient information to identify individual patients across cohorts, potential overlap between reported series could not be definitively excluded, particularly in relation to the multicenter survey, which included two authors who also contributed to two other included studies. Therefore, the total of 4937 reported patients should not be interpreted as a confirmed count of unique individuals.

Among the included studies, 14 (56%) were retrospective case series, 4 (16%) were case reports, three (12%) were case series with unspecified design, one (4%) was a comparative cohort study, one prospective study (4%) and two (8%) were surgeon surveys.

The included studies varied widely in sample size, ranging from single-patient case reports to a multicenter survey including 2226 patients. Across the included studies, the reported patient age ranged from 16 to 65 years, and both male and female patients were included. Among the studies reporting patient sex, 47 were male. The studies included were published between 2003 and 2026.

Implant placement was heterogeneous across the included studies. 18 studies exclusively used the intramuscular plane (IM) (two reported endoscopically assisted technique, one with modified technique of wound management, and four with XYZ technique). Three exclusively used the submuscular plane (SM) (one endoscopically assisted), and one reported the use of both IM and SF implant planes. Two (one case reports and one case series of two patients) didn’t report this information. One surgeon-survey reported that 13 of 19 surgeons preferred the IM plane but did not specify the implant plane used for individual patients, precluding inclusion in the overall distribution of implant location.

Excluding the multicenter survey by Mofid et al.^23^ (2226 patients), for which patient-level implant plane distribution was not available, as well as studies that did not report implant plane, among patients from the remaining studies, 2337 received intramuscular implants, 225 received submuscular implants, and 154 received subfascial implants.

Several studies reported technical modifications of the surgical approach. Badin et al.^41^ and Ibarra-Hurtado^46^ used endoscopic assistance, while Danilla et al. ,^32^ Gonzalez et al. ,^33^ Azevedo et al.^50^ and Castilho et al. ,^52^ used a specific modified technique (XYZ technique). These modifications were described as variations of implant placement technique. Salamat et al.^37^ described a modified wound management technique using retention sutures along the flap closure with the aim of reducing postoperative infection rates.

Implant characteristics were inconsistently reported across studies. Seven studies didn’t report this information. The remaining studies frequently reported only selected features, such as implant surface (smooth vs textured), shape (round vs oval/anatomical), or filler material (silicone gel vs elastomer), while complete characterization of implants was rarely available. Therefore, a pooled analysis of implant type was not feasible, and no clear predominance of a specific implant type could be identified due to incomplete reporting.

Infection incidence could not be reliably pooled due to heterogeneous definitions, incomplete reporting, and variable follow-up duration.

Details regarding infection characteristics, including clinical presentation and time to onset, were available only in a limited number of studies. Treatment approaches were also heterogeneously reported, preventing a pooled analysis of infection management strategies.

Microbiological findings were reported in five studies. Senderoff et al.^21^ reported 11 infections caused by *S. aureus* and one caused by *Escherichia coli*. Ramirez-Sanchez et al.^40^ reported two cases of infection due to *Cutibacterium avidum*. Alperovich et al.^44^ reported one case of late infection caused by *Pseudomonas aeruginosa*. Stojicic et al.^47^ reported one case of necrotizing fasciitis caused by *E. coli*. Landfalf et al.^54^ reported one case of early infection caused by a *Coagulase-negative Staphylococcus.*

Two additional studies (Gonzalez et al. and Hidalgo et al.^33^^,^^49^) reported that antibiotic therapy was tailored according to microbiological culture results; however, the isolated organisms and corresponding microbiological findings were not specified.

Regarding the association between implant plane and infection, only one study provided stratified data. Senderoff et al.^21^ reported 3 infections in the intramuscular (IM) group (3/46, 6.5%) and 10 infections in the subfascial (SF) group (10/154, 6.5%). Serra et al.^48^ stratified their population into two groups according to the surgical technique: group A included 20 patients who underwent gluteoplasty with a modified technique, whereas group B included 20 patients treated with the classic technique. They reported no infections in group A and 1 infection in group B (0%vs 5%, respectively).

Regarding the association between implant filler material and infection, only one study provided stratified data. Hidalgo et al.^49^ reported three infections among patients receiving silicone gel implants (3/75, 4.0%) and no infections among patients receiving silicone elastomer implants (0/22, 0%).

Three studies reported the use of pocket/implant irrigation with antiseptic or antibiotic solutions. Senderoff et al.^21^ used a povidone-iodine solution and reported 13 infections among 200 patients, whereas Andrade et al.^42^ used a combination of cefazolin and gentamicin and reported no infections in a small cohort of 12 patients. Mofid et al.^23^ CIT reported intraoperative irrigation practices among surveyed surgeons; however, the type of irrigation solution was not specified.

When reported, treatment strategies included antibiotic therapy and implant removal. For wound infections considered superficial by the reporting authors, conservative treatment was adopted, allowing implant retention. One study (Hidalgo et al.^49^) reported delayed reimplantation, with a new implant placed 6 months after resolution of the infection.

Follow-up duration was inconsistently reported across studies. Among the studies providing follow-up data (n = 8), mean follow-up ranged from 6 to 32 months.

### Methodological quality

The methodological quality of the included studies was variable. According to the Joanna Briggs Institute (JBI) Critical Appraisal Checklists, 15 studies (60%) were classified as low risk of bias (quality score ≥70%), six studies (24%) as moderate risk of bias (quality score 50–69%), and four studies (20%) as high risk of bias (quality score <50%). The most frequent limitations were related to retrospective study design, unclear reporting of consecutive inclusion, incomplete description of follow-up, and lack of standardized outcome assessment.

### Certainty of evidence

The certainty of evidence was rated as low for infection incidence and very low for the remaining three prespecified outcome domains: pathogen distribution, implant plane and infection, and management of infection. Downgrading was considered across the five GRADE domains—risk of bias, inconsistency, indirectness, imprecision, and publication bias—according to the specific limitations identified for each outcome. Detailed GRADE judgements and the rationale for each domain are presented in Table 2.

**Table 2:** GRADE evidence profile.

Outcome	No. of studies	Risk of bias	Inconsistency	Indirectness	Imprecision	Publication bias	Overall certainty
Infection incidence	25	Not serious	Not serious	Not serious	Not serious	Not serious	Low
Pathogen distribution	5	Not serious	Not serious	Not serious	Serious^b^	Not serious	Very low
Implant plane and infection	2	Serious^a^	Not serious	Not Serious	Serious^b^	Not serious	Very low
Management of infection	10	Serious^a^	Not serious	Not serious	Serious^b^	Not serious	Very low

a Downgraded for risk of bias due to methodological limitations of the included studies.

b Downgraded due to the limited number of studies and/or events.

## Discussion

The present systematic review summarizes the currently available evidence on infectious complications following implant-based gluteoplasty. Postoperative infections were reported in a limited number of the included studies. However, the available evidence did not allow reliable estimation of infection incidence because infectious outcomes were inconsistently reported, two included studies consisted of surgeon surveys without sufficiently detailed patient-level data, and definitions and follow-up were heterogeneous across studies. Importantly, infection frequencies reported in individual studies should not be interpreted as estimates of the population-level incidence, given the heterogeneity in study design, denominators, follow-up duration, definitions of infection, and methods of outcome ascertainment. Nevertheless, when infections occurred, they were associated with clinically significant consequences, frequently requiring implant removal.

From a clinical perspective, although the available evidence suggests that postoperative infections were infrequently reported in the included literature, surgeons should stay vigilant when assessing patients who experience ongoing pain, wounds complications, or slow healing after gluteal implant augmentation., as delayed diagnosis could lead to the need for implant removal and additional surgeries.

Furthermore, the available evidence was insufficient to determine whether implant characteristics, surgical plane, or technical modifications significantly have a significant impact on the risk of infection. Although some studies provided stratified data according to implant plane or filler material, these analyses were limited to isolated reports and did not allow definitive conclusions. Several technical modifications have been proposed^32^^,^^33^^,^^37^ to optimize implant positioning and reduce postoperative complications. However, available evidence regarding their effect on infection risk remains insufficient, as these approaches have mainly been evaluated in small retrospective series or case reports without appropriate comparative groups. In the only study providing stratified data,^21^ infection rates appeared similar between intramuscular and subfascial placement (6.5% in both groups), which were the two most frequently reported implant planes among the included studies. However, these findings should be interpreted cautiously due to the limited number of studies providing comparative information. Therefore, the available evidence does not support a preferential implant plane based on infection risk alone.

Preventive strategies, such as intraoperative pocket irrigation with antiseptic or antibiotic solutions, were reported in only three studies.^21^^,^^23^^,^^42^ The limited available data does not allow conclusions regarding their effectiveness in reducing infection risk.

Microbiological data were rarely reported. Among the limited number of infections with available microbiological confirmation, *Staphylococcus aureus* was the most frequently identified organism, although the limited number of reported cases prevents reliable estimation of pathogen distribution. The predominance of *Staphylococcus aureus* is consistent with its established role as the leading pathogen in implant-related surgical-site infections in plastic surgery,^55^, ^56^, ^57^ although the limited microbiological reporting precludes firm conclusions.

Management strategies varied across studies and included antibiotic therapy, implant removal, and, in selected cases, conservative treatment with implant retention. However, since the severity of infections and the reasons for treatment were reported inconsistently, the optimal management approach cannot be determined from the current evidence. Delayed implant replacement after infection resolution was reported in isolated cases, suggesting that reimplantation could be a viable option for certain patients, but overall, the evidence is still quite limited.

The methodological quality assessment showed that a substantial proportion of included studies met the predefined criteria for low risk of bias according to the JBI checklists; however, the overall certainty of evidence remains limited. This is mainly due to the predominance of retrospective case series and case reports, with only limited comparative cohort evidence without control groups, together with heterogeneity in study design, surgical techniques, implant planes, follow-up duration, and outcome reporting. These limitations also prevented quantitative synthesis through meta-analysis, as infection rates and risk factors could not be meaningfully pooled across studies.

The GRADE assessment indicated low certainty of evidence for infection incidence and very low certainty for the remaining three outcome domains, mainly due to the observational nature and methodological limitations of the available studies, as well as imprecision and limited comparative data. Therefore, the findings should be interpreted cautiously and considered primarily descriptive.

The main limitation of the available literature lies in the lack of standardized reporting of infectious complications. Several studies did not provide a clear definition of infection severity, timing of onset, microbiological findings, or criteria for implant removal. This variability significantly limits comparisons between surgical approaches and hampers the identification of potential risk factors. Moreover, the absence of standardized outcome definitions and the scarce comparative data prevent reliable assessment of causal links between surgical factors and infection risk. Notably, the largest included series by Mofid et al^23^ comprising 2226 patients, offered valuable insight into overall surgical experience; however, the way implant plane distribution was reported—based on surgeon preference rather than individual patients—limits its usefulness for exploring the connection between implant plane and infection risk.

This review has limitations. First, the inclusion of studies with different designs and reporting standards reflects the current state of the literature but may have introduced additional variability in the synthesis of findings. Second, although a comprehensive search strategy was applied across multiple databases, the possibility of publication bias cannot be excluded, as unusual or severe infectious complications may be more likely to be reported than uncomplicated postoperative courses. Additionally, two included studies were surgeon surveys. Although they provided useful information regarding surgical practice and complication patterns, the level of detail was insufficient to allow extraction of patient-level outcomes, and therefore these studies were not suitable for quantitative assessment of infection frequency. The exclusion of secondary gluteoplasties, which was predefined in the eligibility criteria because revision procedures may be associated with a potentially higher risk of infectious complications, limits the generalizability of our findings to primary gluteoplasty procedures. Therefore, the infection rates identified in this review should not be extrapolated to patients undergoing secondary or revision surgery, in whom the risk profile may differ. A further limitation is the potential overlap between patient cohorts reported across the included studies, which should be considered when interpreting the overall sample size. Although 4937 patients were reported, this number may overestimate the number of unique individuals, particularly given the large multicenter survey. This should be taken into account when interpreting the overall burden of reported infections.

Taken together, the current evidence suggests that postoperative infection after gluteal implant augmentation is a potentially severe complication, although its true frequency remains uncertain, and high-quality comparative evidence is still lacking.

Future studies should adopt standardized definitions of infection-related outcomes and report patient characteristics, implant features, microbiological findings, time of onset, infection severity and management strategies in a consistent manner. This would allow more reliable assessment of infection rates and identification of modifiable risk factors in implant-based gluteoplasty.

## Conclusions

Postoperative infections following gluteal augmentation with silicone implants appear to be uncommon but clinically significant complications that may result in implant removal and additional surgical procedures. However, the interpretation of available evidence is limited by inconsistent reporting, heterogeneous outcome definitions, and the predominance of retrospective studies with limited comparative data. Current data does not allow definitive conclusions regarding the role of implant characteristics, surgical plane, or preventive measures in influencing infection risk. Further studies with standardized reporting and prospective comparative designs are needed to better define infection rates, clarify potential modifiable risk factors, and guide prevention and management strategies in implant-based gluteoplasty.

## Author contributions

ER designed the study, supervised the whole project. EB performed the study, wrote the article. FS performed the study, wrote the article.

All authors contributed to editorial changes in the manuscript. All authors read and approved the final manuscript.

## Ethics approval and consent to participate

Not required (publicly available data only).

## Funding

None.

## Availability of data and materials

Data/materials are available from the corresponding author upon request.

## Declaration of competing interest

The authors declare no conflict of interest.
